# A community-based survey to assess risk for one health challenges in rural Philippines using a mobile application

**DOI:** 10.1186/s42522-022-00063-0

**Published:** 2022-04-05

**Authors:** Tae Youn Kim, Loinda Baldrias, Sophia Papageorgiou, Edna Aguilar, Michael Tee, Terra Kelly, Jim Hill, Michael Wilkes

**Affiliations:** 1 Betty Irene Moore School of Nursing, University of California Davis, 2450 48th Street, Suite 2600, Sacramento, CA 95817 USA; 2 College of Veterinary Medicine, University of the Philippines, Los Baños, Laguna Philippines; 3 One Health Institute, School of Veterinary Medicine, University of California Davis, Davis, CA USA; 4 College of Agriculture and Food Science, University of the Philippines, Los Baños, Laguna Philippines; 5 College of Medicine, University of the Philippines, Manila, Philippines; 6 College of Agriculture and Environmental Sciences, University of California Davis, Davis, CA USA; 7 School of Medicine, University of California Davis, Sacramento, CA USA

**Keywords:** One Health, Surveillance, Public Health, Risk Factors, Survey, mHealth Technology

## Abstract

**Background:**

Recent emerging and re-emerging diseases in animals and humans show the vulnerability of humans, animals, and crops to disease outbreaks and the large potential impact on health, food security, and economies worldwide. A technology-enabled One Health (OH) surveillance program offers an opportunity for early detection and response as well as prevention of disease outbreaks in resource-limited settings. As an initial step toward developing the surveillance program, we aimed to identify at-risk groups of households for potential shared health challenges at the human-animal-environmental interface in a rural community of the Philippines.

**Methods:**

A cross-sectional household survey was conducted in the municipality of Los Baños in proximity (63 kilometers south) to Metro Manila by enumerators living in the same community. Twenty-four enumerators conducted household interviews asking a) household characteristics including ownership of animals and crops; b) awareness, beliefs and knowledge about OH; c) family-level health practices related to sanitation, hygiene, and food safety; and d) risk factors for potential OH issues. All data collection and transferring process were streamlined using a mobile application.

**Results:**

Of 6,055 participating households, 68% reported having one or more of gardens, farms, and animals for various reasons. While only 2% of the households have heard about OH, 97% believed they can get disease from animals, plants or the environment. A latent class analysis with nine risk factors for potential OH issues suggested that 46% of the households were at moderate to high risk for exposure to zoonotic pathogens and environmental contaminants.

**Conclusion:**

Our findings indicate that there are unaddressed threats to human, animal, and plant health. Given the importance of the interconnections between the health of humans, animals, and plants, further evaluations of the at-risk households would be necessary to mitigate potential shared health threats in the community. Further, our study demonstrates that mHealth technology can provide an opportunity to systematically assess potential one health problems in the rural communities with limited internet connection.

**Supplementary Information:**

The online version contains supplementary material available at 10.1186/s42522-022-00063-0.

## Introduction

As the global human population increases, people are forced to live in closer proximity to both wild and domestic animals, and to share limited food and water supplies, resulting in the exposure and vulnerability to new pathogens and toxins [1]. Nearly 60% of human pathogens are zoonotic and 75% of emerging zoonotic pathogens (e.g., Nipah virus, SARS-CoV-2, WNV, Ebola, Plague, Zika virus) originate in wild animals [2, 3]. However, it is not only zoonotic pathogens that pose a threat to global health [4]. For centuries infectious diseases have passed back and forth between humans and animals often mitigated by external factors, including cultural practices, agriculture, climate, socioeconomic factors, geography, and dietary patterns. Human behavior changes related to lifestyle, animal husbandry, agricultural practices, community planning and home sanitation also have a large impact on health and disease worldwide.

Continuous monitoring and surveillance of newly emerging/re-emerging diseases and pandemic outbreaks is a critical component of One Health (OH) which is the term representing the collaboration of multiple disciplines working locally, nationally, and globally to achieve optimal health for people, animals, plants/crops, and the environment [2, 5]. A technology-enabled community OH surveillance program can greatly assist in early detection and prevention of outbreaks in resource-limited settings [6]. In addition to infectious diseases, OH is concerned with insect vectors transmitting diseases, and environmental contamination such as waste dumped in or near water systems [7], affecting crops, and animal and human communities [8, 9]. Diseases and insect infestations in plants and crops emerge due to the importation of non-native and invasive plant or crop species, introduction of novel pests, or as a result of adverse environmental impacts.

These shared health threats often result in a substantial economic burden on individuals (mortality and morbidity with resultant loss of income), families, communities, and entire nations [10, 11]. In resource constrained regions, these economic impacts are particularly significant given high population density and growth rates, lack of sanitary improvements, food insecurity, limited access to agricultural “best practices” and suboptimal health care infrastructure. Given the globalization over the past 20 to 30 years and the ability of pathogens to rapidly spread, an integrated OH approach is paramount to identify and minimize the impact of diseases worldwide and agricultural pestilence.

### Establishment of a research partnership in the Philippines

The Philippines, with a population of nearly 111 million people, spends approximately 4% of its gross domestic product on healthcare [12]. Health care systems in the Philippines are overburdened and under-resourced with a shortage of well-trained and equitably deployed health workers, particularly in geographically isolated rural regions where 53% of the population resides [13]. Large regions of the country have limited access to primary care and even fewer have adequate access to expertise or services for animal or plant health, limiting early identification of health threats. Importantly, there is little data on current OH practices (infectious disease control, food safety, sanitation, potable water hygiene, agricultural pesticide use, animal husbandry, etc.) at the local level, making it very difficult for policy makers to effectively allocate resources and provide evidence-based interventions to health professionals. Low cost, sustainable approaches directed at grassroot community-based interventions can play an important role in mitigating the effects of some common causes of morbidity and mortality, as well as agricultural disease and pests leading to food insecurity [6].

Accordingly, a research partnership between the University of California Davis and the University of Philippines (Diliman, Los Baños, and Manila) was established in March 2017 with the goal of improving the health of humans, animals, plants, and the environment in the Philippines. This multi-disciplinary research team included experts from human medicine, nursing, veterinary medicine, agriculture, health informatics, and computer science. As an initial step toward developing a technology-enabled surveillance program with a user-friendly data collection tool [6], this OH project conducted a cross-sectional survey of households in rural communities of the Philippines. The purpose of this survey was to 1) describe demographic characteristics of participating households, including ownership of animals, farms or gardens, 2) examine participants’ awareness, beliefs and knowledge about OH issues (related to human, animal, agriculture, and environmental health), 3) examine family-level health practices related to sanitation, hygiene, and food safety, 4) assess prevalence of risk factors for potential OH issues, and 5) identify clusters of households at high risk for health challenges at the human-animal-environmental interface.

## Methods

**Fig. 1:**
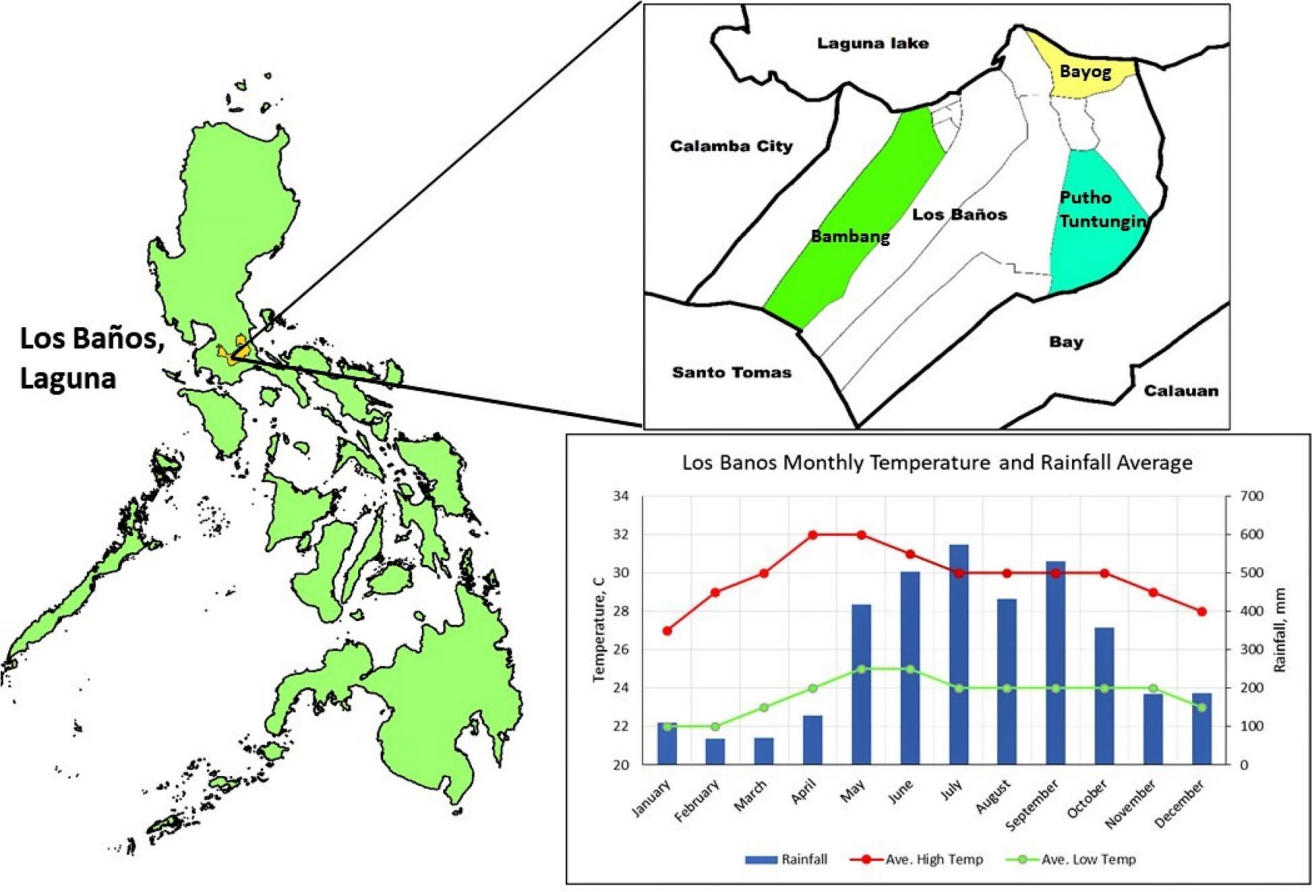
Geographic Location of Barangays Bambang, Bayog and Tuntungin-Putho. *Note*: A barangay (village) is considered the smallest administrative district in the Philippines. The bar graph presents the average monthly rainfall while the line graph shows the average monthly high (red line) and low (green line) temperature in Los Baños, Laguna, Philippines

The survey was administered between January 2018 and March 2018 in the broader municipality of Los Baños on the island of Luzon, Laguna, Philippines. The municipality has a tropical monsoon climate and consists of 14 barangays (villages) of which three were chosen in this study based on their geographical, economic, and ecological diversity. Figure 1 shows the geographic location of Barangays Bambang, Bayog, and Tuntungin-Putho. The project was officially approved by the Institutional Review Board of the University of California Davis and the Research Ethics Board of the University of the Philippines Manila.

### Training survey enumerators

We developed a household survey questionnaire running on a mobile application (OH App) to streamline remote data collection. Five enumerators were initially trained on the use of the OH App and how to conduct the survey. Field testing was then conducted through a mock survey in the three rural villages (Fig. 1). Mock survey results were discussed with the enumerators and community member-volunteers. Feedback on the enumeration process and the OH App was collected from the enumerators, along with direct observations by project staff and the software developers. All comments were then reviewed by the research team for improvements of the survey questionnaire, enumeration process, and the OH App prior to the start of a community-based survey.

After the survey questions were finalized, a user manual was written for survey enumerators detailing how to use the App including data synchronization and how to administer the survey in different household environments. Twenty-four enumerators were recruited from the three barangays, oriented and trained for the final survey administration, which included utilizing role playing among the enumerators and field testing in an adjacent barangay Maahas. Barangay leaders met with the research team and permission to conduct the survey was obtained from elected officials in each barangay. Field testing included four households per enumerator pair and an assessment exercise to assure accurate data collection. The enumerators were provided with an electronic tablet installed with the OH App, the user manual, hard copies of the survey questionnaire (should the App or tablet fail in the field), and OH informational flyers and foldable fans as a small token of appreciation for participating households. All enumerators wore t-shirts with the project’s logo to identify them properly in the community.

### Survey administration

The 24 enumerators were divided into three groups – 10 enumerators for Barangay Bayog, six for Barangay Tuntungin-Putho and eight for Barangay Bambang, in proportion to the household population of the three barangays. Trained enumerators conducted structured household interviews using the OH App with the survey questionnaire (see Additional file 1). Data collected on the mobile devices were encrypted and sent to a secure cloud server when internet connection was established in the field. Regular meetings were conducted with the enumerators to address issues regarding survey administration, field conditions, and survey progress. Household interviews were monitored by local project field team members for quality and accuracy. The field team observed enumerators on random household visits and the project staff conducted regular quality control assessment of the survey data.

### Study measures

Respondents to the survey were asked to provide 1) household characteristics such as demographics and ownership of animals and crops, 2) awareness, beliefs and knowledge about OH, 3) family-level health practices related to sanitation, hygiene, and food safety, and 4) risk factors for potential OH issues.

1. *Household characteristics*: Respondents provided their age, sex (male/female), education (elementary, high school, college, advanced degree), number of household members, average monthly income of household (low, middle, high income), major provider of medical care (community health center, doctor’s private office, hospital outpatient clinic, others), and residence location (village 1, 2 or 3).

Additionally, we asked about crops planted on their property that might constitute a family vegetable or fruit garden/farm and/or a commercial garden/farm. Crops were classified into four categories (vegetables, grains, fruits, and ornamentals) and their purpose was coded as household consumption, commercial productions or other. Likewise, each participating household was asked about their animal ownership. Animals were categorized as domestic carnivores (dogs, cats), domestic fowl (chickens, turkeys, ducks/geese), ruminants (goats, cattle/buffalo), pigs/swine, birds, and others. The purposes of having these animals were coded as household food consumption, pet/companion, sale as food, breeding for sale, and others.

2) *One Health awareness, beliefs, and knowledge*: The awareness of the OH concept and OH issues in their local environment was assessed by asking whether they had heard the term One Health (yes/no) and whether they were aware of any contaminants affecting their crops and/or animal drinking water over the past year, including insecticides, microorganisms, heavy metals, and rodenticides. Each contaminant chosen was coded as 1 (yes) or 0 (no). OH beliefs were assessed using one multiple choice question “Do you believe that humans can get diseases from animals, plants, and/or environment?” Respondents’ knowledge about OH was assessed using 15 true/false questions and each correct answer was given a 1-point. The OH knowledge score ranged from 0 to 15 points, which was computed by summing the total number of correct answers.
3) *Family-level health practices*: A 12-item questionnaire was used to evaluate household practices related to sanitation, hygiene, and food safety. Each low-risk behavior performed in the past year was coded as ‘1’ and then summed for a total score (range: 0-12 points).
4) *Risk factors for potential OH issues*: The respondents were asked for potential exposure to environmental contaminants and zoonotic pathogens.
  4.1 *Use of fertilizers/pesticides*. Households with a farm or a garden were asked about their use of fertilizers (yes/no) and pesticides (yes/no) in the past year, as well as causes of crop loss, if any. Multiple options were provided for known causes of crop loss, including extreme weather event (such as typhoon, drought), raiding or contamination by wild animals (such as pests/rodents/birds), plant diseases (caused by fungi, viruses, nematodes, bacteria, etc.), and others.
  4.2 *Presence of threats to animal health*. Households having animals were further asked whether they observed potential health threats to their animals. Multiple choice options included a lack of vaccinations, limited accessibility to veterinary care, limited knowledge or training on how to handle animals, poor housing of animals, and others. We also asked whether their animals had adequate access to feed/forage (yes/no).
  4.3 *Animal slaughter and contact with wild animals*. We asked one yes/no question regarding animal slaughter (“Are animals slaughtered / butchered at your home?”) and one multiple-choice question assessing their contact with rodents, snakes/reptiles, fish, and others. Types of contact with these wild animals were coded as entering living space, hunting, pet/companion, and others.
  4.4 *Prevalence of household insects*. Survey participants were asked whether they observed any insects around their household and/or farm. Multiple choice options included mosquitoes, cockroaches, flies, and others (e.g., mites, ticks, and fleas). Each response was coded as 1 (yes) or 0 (no).

### Statistical analysis

The unit of analysis was a household in this study. All study variables were summarized using descriptive statistics including frequencies/proportions for categorical variables and means/standard deviations for continuous variables. Bivariate analysis included a Spearman’s correlation, Chi-square tests, and one-way analysis of variance (ANOVA) for means comparison. Latent class analysis (LCA) was conducted using the poLCA package [14] in R environment [15] to fit a set of clusters representing the distinct prevalence of nine risk factors while controlling for the covariates – the geographic location of three villages and ownership of animal/farm/garden. The selection of best clusters was completed according to the commonly used model selection criteria, including Bayesian information criterion (BIC), Akaike information criterion (AIC), and the likelihood ratio χ2 statistic for goodness of fit [14]. A significance value of 0.05 was used throughout the analysis.

## Results

Average survey time was 35 minutes per household. A total of 6,055 households voluntarily participated in this survey. The majority of respondents were male (56%) with a mean age of 45.9±14.7 years old and high school graduate (50%). The proportion of respondents with educational attainment of some college education or above was approximately 29%. Overall the educational attainment of the respondents was much higher than a median of 10 years of schooling in the adult population [16].

### Characteristics of participating households

Of 6,055 households, 75% (*n* = 4,568) were considered low-income households below the national average family income of 21,600 pesos/month ($453/month). The average number of individuals in a household was 3.9±1.9 (range: 1~14). The most common locations where families sought medical care was at the Barangay community health centers (*n* = 2,633, 44%) followed by private doctor’s offices (*n* = 1,217, 20%) and hospital outpatient clinics (*n* = 1,187, 19.6%).

As shown in Table 1, comparisons across the three villages show important differences. Village 1 represented 28% (*n* = 1,701) of the cohort and resided in an area focused on agricultural (rice) development in Los Baños. While 35% (*n* = 2,094) lived in village 2 which is located at the foot and slopes of the volcano, the remaining 37% (*n* = 2,260) of the households resided in village 3 situated near the lake of a dormant volcano. Two thirds of the households (*n* = 4,101, 68%) owned animals, gardens and/or farm for various purposes, including sales and household consumption (Tables 2 and 3). Overall households residing in village 3 were more likely to own farms, gardens, and animals compared to those in the other two villages (*p* < .001). Also, households in village 3 were more likely to grow crops and raise animals for sale than those in villages 1 and 2 (*p* < .001).

**Table 1:** Characteristics of households participated in the survey (*n* = 6,055 households)

**Characteristics**	**Total** **n (%)**	**Village 1** **n (%)**	**Village 2** **n (%)**	**Village 3** **n (%)**	**P-value**
**Number of households surveyed**					
Number of persons per household*, Mean±SD	3.9±1.9	3.5±1.8	3.7±1.7	4.3±1.9	< 0.001
Low income (< 21,600 peso/month)	4,568 (75.4)	1,236 (72.7)	1,705 (81.4)	1,627 (72.0)	< 0.001
**Medical care**					< 0.001
Community health centers	2,663 (44.0)	1,030 (60.6)	685 (32.7)	948 (41.9)	
Doctor’s private office	1,217 (20.1)	445 (26.2)	471 (22.5)	301 (13.3)	
Hospital outpatient clinic	1,187 (19.6)	59 (3.5)	492 (23.5)	636 (28.1)	
**Ownership of animal/farm/garden**					
Households without animal/farm/garden	1,954 (32.3)	732 (43.0)	628 (30.0)	594 (26.3)	< 0.001
Households with farm/garden	2,657 (43.9)	542 (31.9)	932 (44.5)	1,183 (52.3)	< 0.001
Households with animals	3,239 (53.5)	774 (45.5)	1,144 (54.6)	1,321 (58.5)	< 0.001

*Note:* Village 1 – resided in an area focused on agricultural (rice) development

Village 2 – located at the foot and slopes of the volcano

Village 3 – situated near the lake of a dormant volcano

SD = standard deviation

**Table 2:** Types and purposes of growing crops (*n* = 2,657 households)

**Number of households with farm/garden**	**Total** **n = 2,657**	**Village 1** **n = 542**	**Village 2** **n = 932**	**Village 3** **n = 1,183**	**P-value**
	**n (%)**	**n (%)**	**n (%)**	**n (%)**	
**Types of crops**					
Grains	42 (1.6)	9 (1.7)	12 (1.3)	21 (1.8)	n.s
Ornamentals	2,064 (77.7)	346 (63.8)	733 (78.6)	985 (83.3)	< 0.001
Vegetables	1,341 (50.5)	309 (57.0)	446 (47.9)	586 (49.5)	< 0.001
Fruits	1,606 (60.4)	332 (61.3)	618 (66.3)	656 (55.5)	< 0.001
**Purpose**					
Household consumption	1,821 (68.5)	444 (81.9)	783 (84.0)	594 (50.2)	< 0.001
Commercial production	406 (15.3)	14 (2.6)	109 (11.7)	283 (23.9)	< 0.001
**Risk factors for One Health issues**					
Loss of crops	796 (30.0)	133 (24.5)	122 (13.1)	541 (45.7)	< 0.001
Use of fertilizers	517 (19.5)	11 (2.0)	117 (12.6)	389 (32.9)	< 0.001
Use of pesticides	289 (10.9)	10 (1.8)	33 (3.5)	246 (20.8)	< 0.001

*Note:* n.s. = not significant

**Table 3:** Types and purposes of having animals (*n* = 3,239 households)

**Number of households with animals**	**Total** **n = 3,239**	**Village 1** **n =774**	**Village 2** **n = 1,144**	**Village 3** **n = 1,321**	**P-value**
	**n (%)**	**n (%)**	**n (%)**	**n (%)**	
**Types of animals**					
Domestic carnivores: Dog, cat	2,876 (88.1)	684 (88.4)	1,028 (89.9)	1,164 (88.1)	0.561
Domestic fowl: Chicken, turkey, duck	747 (23.1)	177 (22.9)	229 (20.0)	341 (25.8)	0.009
Ruminants: Goat, cattle	49 (1.5)	4 (0.5)	28 (2.4)	17 (1.3)	0.006
Pig/swine	48 (1.5)	3 (0.4)	23 (2.0)	22 (1.7)	0.032
Birds	147 (4.5)	29 (3.7)	33 (2.9)	85 (6.4)	< 0.001
**Purpose**					
Pet/companion	2,974 (91.8)	731 (94.4)	1,099 (96.1)	1,144 (89.6)	< 0.001
Household consumption	239 (7.4)	47 (6.1)	70 (6.1)	122 (9.2)	0.010
Breeding for sale	230 (7.1)	63 (8.1)	62 (5.4)	105 (7.9)	0.055
Sale as food	168 (5.2)	33 (4.3)	41 (3.6)	94 (7.1)	< 0.001
**Risk factors for One Health issues**					
Home slaughter	291 (9.0)	46 (5.9)	65 (5.7)	180 (13.6)	< 0.001
Presence of animal health threats	2,031 (62.7)	316 (40.8)	891 (77.9)	824 (62.4)	< 0.001

### One Health awareness, beliefs, and knowledge

While only 132 households (2.2%) indicated that they have heard the term ‘One Health’ prior to the survey, many had fundamental knowledge of core OH concepts related to promoting health at the human-animal-environmental interface. For example, 97% of the respondents believed humans can get disease from animals, plants, or the environment. Further 35% of the respondents indicated they were aware of one or more contaminants, such as insecticides and rodenticides, affecting crops or animal drinking water over the past year. Using the 15 questions assessing OH knowledge, 48% of the respondents had 11 or more correct answers, 34% of the respondents had 6-10 correct answers, and the remaining 18% presented a minimal understanding with five or fewer correct answers (Table 4).

**Table 4:** One Health awareness, beliefs, and knowledge (*n* = 6,055 households)

**Characteristics**	**Total** **n = 6,055**	**Village 1** **n = 1,701**	**Village 2** **n = 2,094**	**Village 3** **n = 2,260**	**P-value**
	**n (%)**	**n (%)**	**n (%)**	**n (%)**	
Have heard the term One Health (OH)	132 (2.2)	31 (1.8)	42 (2.0)	59 (2.6)	n.s
Awareness of any contaminants (e.g., insecticides, microorganisms, heavy metals, and rodenticides) affecting crops or animal drinking water over the past year	2,118 (35.0)	1,053 (61.9)	985 (47.0)	80 (3.5)	< 0.001
OH Beliefs: Human can get disease from 1) animals, 2) plants, and 3) environment					< 0.001
Number of correct answers: 0	206 (3.4)	140 (8.2)	19 (0.9)	47 (2.1)	
Number of correct answers: 1-3	5,849 (96.6)	1,561 (91.8)	2,075 (99.1)	2,213 (97.9)	
OH Knowledge score (0-15), Mean±SD	9.7 ± 3.7	7.5 ± 4.7	11.1 ± 2.7	10.1 ± 3.1	< 0.001
No. of correct answers (≥ 11)	982 (16.2)	772 (45.4)	52 (2.5)	158 (7.0)	< 0.001
No. of correct answers (6-10)	2,084 (34.4)	427 (25.1)	785 (37.5)	872 (38.6)	< 0.001
No. of correct answers (≤ 5)	2,914 (48.1)	470 (27.6)	1,247 (59.6)	1,197 (53.0)	< 0.001

*Note:* SD = standard deviation, n.s = not significant

### Family-level health practices related to sanitation, hygiene, and food safety

Of the 6,055 households, 41% (*n* = 2,470) presented having low-risk practices for all 12 practices assessed (Table 5). On the other hand, 44% of the households reported 1 or 2 high-risk practices while the remaining 15% showed 3-7 risky practices. A correlation analysis indicated a weak but significant relationship between OH knowledge and family practices (Spearman’s *rho* = .36, *p* < .01). This means that when the respondents had a better understanding of OH, they were likely to employ safe methods in food preparation and reducing potential exposure to zoonotic pathogens at home. Interestingly, households in village 1 are less likely to own animals, farms or gardens (*p* < .001) and presented the lowest average scores in both OH knowledge and family practices compared to those in villages 2 and 3 (*p* < .001).

**Table 5:** Family-level health practices performed in the past year (*n* = 6,055 households)

**Family Practice**	**Total** **n = 6,055**	**Village 1** **n = 1,701**	**Village 2** **n = 2,094**	**Village 3** **n = 2,260**	**P-value**
	**n (%)**	**n (%)**	**n (%)**	**n (%)**	
Behavior score (0-12), Mean±SD	10.8 ± 1.3	10.2 ± 1.0	11.1 ± 1.4	11.1 ± 1.1	< 0.001
No. of low-risk behaviors (12)	2,470 (40.8)	145 (8.5)	1,328 (63.4)	997 (44.1)	< 0.001
No. of low-risk behaviors (10-11)	2,683 (44.3)	1,207 (71.0)	418 (20.0)	1,058 (46.8)	< 0.001
No. of low-risk behaviors (≤ 9)	902 (14.9)	349 (20.5)	348 (16.6)	205 (9.1)	< 0.001

*Note:* SD = standard deviation

### Prevalence of risk factors affecting One Health issues

As shown in Tables 2, 3, and 6, there were significant variations across the villages in the prevalence of potential One Health problems in the community, including use of fertilizers and pesticides, threats to animal health, animal home slaughter, and contact with wild animals and household insects.

**Table 6:** Prevalence of household insects and contact with wild animals (*n* = 6,055 households)

	**Total** **n = 6,055**	**Village 1** **n = 1,701**	**Village 2** **n = 2,094**	**Village 3** **n = 2,260**	**P-value**
	**n (%)**	**n (%)**	**n (%)**	**n (%)**	
**Any contact with wild animals**	1,817 (30.0)	411 (24.2)	312 (14.9)	1,094 (48.4)	< 0.001
Rodents	1,541 (25.5)	355 (20.9)	274 (13.1)	912 (40.4)	< 0.001
Others (snakes, fish, etc.)	872 (14.4)	200 (11.8)	71 (3.4)	601 (26.6)	< 0.001
**Type of contact with wild animals**					
Enters living space	1,294 (21.4)	327 (20.9)	300 (13.1)	667 (40.4)	< 0.001
Hunting	340 (5.6)	2 (0.1)	1 (0.0)	337 (14.9)	< 0.001
**Prevalence of any household insects**	3,118 (51.5)	744 (43.7)	1,098 (52.4)	1,276 (56.5)	< 0.001
Mosquitoes	2,871 (47.4)	718 (42.2)	926 (44.2)	1,227 (54.3)	< 0.001
Cockroaches	2,213 (36.5)	655 (38.5)	640 (30.6)	918 (40.6)	< 0.001
Flies	2,046 (33.8)	544 (32.0)	637 (30.4)	865 (38.3)	< 0.001
Others (ticks, fleas, mites, lice)	1,097 (18.1)	178 (10.5)	389 (18.6)	530 (23.5)	< 0.001

#### Use of fertilizers and pesticides

A total of 2,657 households reported having a farm or a garden among the three villages (Table 2). Of these, 20 and 11% reported they used fertilizers and pesticides in the past year respectively, while 30% (*n* = 796) reported crop losses due to extreme weather events (e.g., typhoon, drought), wild animals (pests, rodents), or unknown causes in the past year.

#### Threats to animal health

Of the 3,239 households with animals, 42% (*n* = 1,426) expressed concern about inadequate access to feed/forage for their animals. Approximately two thirds of the households (*n* = 2,032, 63%) reported potential health threats to their animals (Table 3). The major health threats included a lack of vaccinations (*n* = 1,088, 54%), limited accessibility to veterinary care (*n* = 797, 39%), limited knowledge or training on how to handle animals (*n* = 666, 33%) and poor housing for animals (*n* = 634, 31%).

#### Animal slaughter

Nine percent (*n* = 291) of the 3,239 households with animals reported that they performed animal slaughter at home, which was more prevalent in village 3 as well (*p* < .001).

#### Contact with wild animals

Thirty percent (*n* = 1,817) of the participating households indicated some type of contact with wild animals across the three villages (Table 6). The most frequently reported wild animals were rodents (*n* = 1,541, 85%) followed by snakes/reptiles (*n* = 797, 44%), and fish (*n* = 261, 14%). Intrusion of these wild animals in personal living spaces constituted the major reason for contact (*n* = 1,294, 71%) followed by hunting (*n* = 340, 19%). Also, wild animals were more frequently observed in village 3 than in the other two villages (*p* < .001).

#### Prevalence of household insects

As shown in Table 6, less than half of the respondents reported mosquitoes (*n* = 2,871, 47%), cockroaches (*n* = 2,213, 37%), and/or flies (*n* = 2,046, 34%). When mosquitoes were reported, more than 97% of the households also reported prevalence of cockroaches and flies. Further, 18% (*n* = 1,097) of the respondents reported exposures to other insects such as mites, ticks, and fleas. All insects were more frequently observed in village 3 than in the other two villages (*p* < .001).

### Identification of households at risk for health challenges at the human-animal-environmental interface

Considering different profiles of OH risk factors presented in the participating households, we performed LCA to determine households that might be at high risk for OH issues. While controlling for geographic location (i.e., village) of households and ownership of animals/crops, the nine prevalent risk factors were entered to LCA, including contact with rodents, contact with wild animals other than rodents, loss of crops due to environment problems, use of fertilizers, use of pesticides, household insects (mosquitoes), household insects (other than mosquitoes), animal home slaughter, and presence of animal health threats (e.g., lack of vaccinations). This analysis resulted in three distinct clusters which are labeled as low-, moderate-, and high-risk groups for having OH related problems (Table 7).

**Table 7:** A summary of household characteristics by risk group (*n* = 6,055 households)

**Household Characteristics**	**Total** **(n = 6,055)**	**Low risk ** **(n = 2,764)**	**Moderate risk** **(n = 2,651)**	**High risk ** **(n = 640)**	**P-value**
	**n (%)**	**n (%)**	**n (%)**	**n (%)**	
**Geographic location**					< 0.001
Reside in village 1	1,701 (28.1)	950 (34.4)	750 (28.3)	1 (0.2)	
Reside in village 2	2,094 (34.6)	960 (34.7)	1,096 (41.3)	38 (5.9)	
Reside in village 3	2,260 (37.3)	854 (30.9)	805 (30.4)	601 (93.9)	
**Ownership of farm, garden and/or animal(s)**					
Ownership of farm/garden	2,657 (43.9)	762 (27.6)	1,297 (48.9)	598 (93.4)	< 0.001
Ownership of animal(s) (e.g., dog, cat, chicken, turkey, duck, goat, etc.)	3,239 (53.5)	92 (3.3)	2,649 (99.9)	498 (78.8)	< 0.001

*Note:* Village 1 – resided in an area focused on agricultural (rice) development

Village 2 – located at the foot and slopes of the volcano

Village 3 – situated near the lake of a dormant volcano

**Fig. 2:**
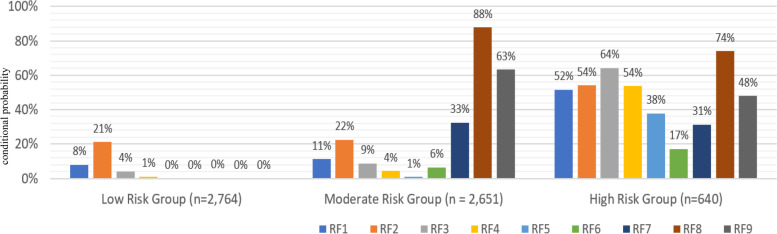
Conditional Probabilities of Having One Health Related Risk Factors (RF) by Group. Note: RF1 = Contact with wild animals; RF2 = Contact with rodents; RF3 = Loss of crops due to environment problems; RF4 = Recent use of fertilizers; RF5 = Recent use of pesticides; RF6 = Animal slaughter at home; RF7 = Household insects (others); RF8 = Household insects (mosquitoes); RF9 = Presence of animal health threats (e.g., lack of vaccinations, poor housing)

As shown in Fig. 2, conditional probabilities of having all but two risk factors are much higher in the high-risk group compared to the other two groups (*p* < .001). The high-risk group involves approximately 11% (*n* = 640) of the households surveyed. The majority in the high-risk group resided in village 3 (94%) and had farms/gardens (93%), as well as animals (79%). In comparison, all but two households in the moderate-risk group (*n* = 2,651) had one or more animals while only less than half of the households owned farms/gardens. Subsequently, 63% of the moderate-risk group addressed the presence of animal health threats in the community. Contrarily, the proportion of households having animals (3%) and farm/gardens (28%) was significantly lower in the low-risk group (*n* = 2,764).

## Discussion

Recent emerging and re-emerging diseases in animals and humans show the vulnerability of humans, animals, and crops to disease outbreaks and the large potential impact on health and food insecurity [17–21]. Our baseline survey analysis clearly indicates potential threats to human, animal, and plant health across the three villages regardless of the geographic location and ownership of animals, farms, or gardens. When at-risk households were further examined for potential OH problems, 11% of the households presented a higher chance of being exposed to both zoonotic pathogens and environmental contaminants. This initial surveillance sheds light on where to allocate resources for active surveillance in the community to leverage mobile health (mHealth) technology and maximize the impact of communicating OH data across the country using a public health information system [6]. For example, if baseline surveillance suggests a subset of a village is more at-risk, then mobile health tools could remind health workers to target those at-risk homes for additional surveillance, health education, or funding to upgrade resources. Importantly, in most rural areas worldwide, the detection of, and response to disease threats or invasive threats to crops and livestock is slow due to 1) limited access to health and agricultural experts, 2) lack of broadly trained community health workforce, and 3) an inadequate technology infrastructure to support data collection, rapid reporting, and intervention.

A technology-enabled community OH surveillance program can greatly assist in the early detection and prevention of a disease outbreak or other outbreaks in resource-limited settings [22]. A first step in designing such community-based technology is to gain an understanding of baseline practices and beliefs around OH – in this case in rural areas of the Philippines. This study showed that within the three rural communities in the Philippines, a mobile device employed by trained enumerators is valuable for collecting household surveillance data on a variety of OH issues. Effective training of enumerators through a combination of didactics and practical training, in conjunction with an easy-to-use, menu driven data collection app probably contributed to the high level of community participation.

Our community-based approach presents a potential pathway to train trusted community health workers in other OH surveillance tasks crucial to enhancing data from rural areas. Often in resource limited regions, funding for data collection is limited and competes with funding for other health care delivery programs. As such, accurate data is given a lower priority and is challenging to acquire. Our menu driven, visually oriented smart phone app was efficient at quickly collecting and aggregating high quality data that could be utilized for rapid response to health threats. We demonstrated that mobile technology relying on cellular technology is affordable, acceptable, and easily learned. The mHealth technology implemented in this study assisted in the data collection process across wide geographic areas that often have limited outreach and network connections, and helped in the compilation and management of data for rapid analysis.

Like most community surveillance assessments, this study had several limitations. One was the availability of the head of household who was often not at home. Further, identifying the “head of household” was difficult to determine so that often the enumerator allowed an adult person to self-designate as head of household. While our enumerators were recruited from the communities being surveilled to enhance public trust, they were selected based upon their application and most had no prior knowledge of OH concepts. Nonetheless, all the enumerators were able to comprehend the issues at hand and OH education was provided efficiently during the training assessment. The enumerators were carefully assessed before they were allowed to collect data in the field. While we were unable to review the quality of all enumerators in our catchment area due to limited resources, random audits by supervisors gave us confidence in our data. Lastly, we used several items to assess knowledge of one health concepts related to safety. The scale derived from these questions has not been validated and this should be done in future research.

The Philippines government, like many others, is faced with important health challenges. Demands for living space, clean water, food security, animal health, crop productivity, and clean, inexpensive energy pose enormous social and economic challenges that have significant impacts on human health and well-being [23]. The goals of the Agriculture and Fisheries Modernization Act (AFMA) are to improve nutrition and healthy food consumption of animals and plants [24], but food safety and security continues to be a concern. This will require providing accessible approaches to safe food production/storage and disease management. Further research is warranted to explore the impact of OH problems on important human-illness oriented outcomes (e.g., infectious diseases, toxic exposures, and the impact of climate change) as well as diseases of animals and crops, and examine the effectiveness of preventive strategies (vaccinations, proper food storage, appropriate pesticide use, well planned sanitation, etc.) in improving the health of humans, animals, and plants.

## Conclusion

Early detection of OH problems and timely interventions have the potential to improve well-being, community engagement, and economic strength in rural regions with limited health resources for humans, animals, and plants. Along with the lack of awareness of OH, the majority of community members faced OH problems in the rural communities of the Philippines, threatening the health of humans, animals, and plants. With a paucity of previous research examining the utility of mobile applications in community-based OH surveillance, our study demonstrated mHealth technology could provide an opportunity to systematically assess current OH practices and beliefs, and potential risk factors for OH problems in the rural communities with limited internet connection. Continuous collaboration among the researchers, providers, administrators, and policy makers will be of importance to bring the full potential of mHealth technology to mitigating recent OH concerns in resource-limited settings.

## Supplementary Information

**Additional file 1.** One Health Survey Questionnaire. A survey questionniare used to collect household data in the community.

## Data Availability

The dataset generated in the study is available from the corresponding author on reasonable request.

## Abbreviations

OH: One Health
AFMA: Agriculture and Fisheries Modernization Act
