# Supplementary material for: A community-based survey to assess risk for one health challenges in rural Philippines using a mobile application
Source: One Health Outlook. 2022 Apr 5;4:7. doi: 10.1186/s42522-022-00063-0 (PMC8979641; doi:10.1186/s42522-022-00063-0)
Supplement: Supplementary file 1 — Additional file 1. One Health Survey Questionnaire. A survey questionniare used to collect household data in the community. [file 42522_2022_63_MOESM1_ESM.docx]

**One Health Survey Questionnaire**

**A. HOUSEHOLD DEMOGRAPHICS**

**Q1. Geographic location**

1. Village 1
2. Village 2
3. Village 3

**Q2. Respondent’s sex**

1. Male
2. Female

**Q3. Respondent’s age:** _____ years

**Q4. Education–highest level completed?**

1. Elementary
2. High school
3. College
4. Advanced degree (MD, DVM, PhD, etc.)

**Q5. Number of household members: ________________**

**Q6. Average household (all members) monthly income?**

1. Low income < 21,600 php
2. Middle income 21,600 – 37,000 php
3. High income > 37,000 php
4. Declines / does not know

**Q7. When you are sick, what are the most common locations that you usually go to seek medical care?**

1. Doctor’s private office
2. Community (barangay) health center or barangay health worker
3. A hospital outpatient clinic or community (barangay) health center
4. Others (specify): _____________________________

**B. OWNERSHIP OF FARMS/GARDENS**

**Q1. Do you own farms / gardens?**

1. Yes (If yes, answer the following questions.)
2. No

**Q2. Which of the followings are planted on your farm or in your garden?** Check all that apply.

1. Vegetables (e.g., string beans, eggplants, tomatoes, cucumbers/squash, etc.)
2. Grains (e.g., rice, corn, mungbean)
3. Fruits (e.g., mango, banana, rambutan, coconut, citrus, etc.)
4. Ornamentals (e.g., cut flowers)

**Q3. What is the purpose of each farm / garden?**

1. Commercial productions
2. Household consumption
3. Other (specify) __________

**Q4. Do you use fertilizers at the farm/garden?**

1. Yes
2. No

**Q5. Do you apply any pesticides to your crops?**

1. Yes
2. No

**Q6. Have you had any crop loss in the past year?**

1. Yes (If yes, what was the cause of the crop loss?)
2. No

**Q7. What was the cause of the crop loss?**

1. Pests / rodents / birds
2. Disease (fungus, virus, Nematodes, bacteria)
3. Extreme weather event (e.g. typhoon, drought, other)
4. Other (specify): _______

**C. OWNERSHIP OF ANIMALS**

**Q1. Do you own animals?**

1. Yes (If yes, answer the following questions.)
2. No

**Q2. What type of the following animals do you own?**

1. Chicken
2. Turkey
3. Duck / goose
4. Goat
5. Cattle / buffalo
6. Dog
7. Cat
8. Pig / swine
9. Other

**Q3. What is the purpose of each type of animals?**

1. Household food consumption
2. Pet / companion
3. Sale as food
4. Breeding for sale
5. Other (specify) __________

**Q4. Are animals slaughtered / butchered at your home?**

1. Yes
2. No

**Q5. What are the biggest threats to the health of your animals?** Please select all that apply.

1. Lack of vaccinations
2. Limited accessibility to veterinary care
3. Limited knowledge or training on how to handle animals
4. Poor housing
5. Other (specify):

**Q6. Do your animals have access to adequate feed / forage?**

1. Yes
2. No

**D. HOUSEHOLD INSETCS & WILD ANIMALS**

**Q1. Which of the following insects have you observed around your household and/or farm?** Please select all that apply.

1. Mosquitoes
2. Cockroaches
3. Flies
4. Other (specify): _______

**Q2. Do you come in contact with any of the following wild animals?** Check all that apply.

1. Rodents
2. Snakes / reptiles
3. Fish
4. Other (specify): _______

**Q3. What type of contact do you have with the wild animals?** Check all that apply.

1. Entering living space
2. Hunting
3. Pet / companion
4. Other (specify): _______

**E. ONE HEALTH AWARENESS, BELIEFS, AND KNOWLEDGE**

**Q1. Have you heard the term “One Health”?**

1. Yes
2. No

**Q2. Are you aware of any contaminants affecting crops or animal drinking water over the past year?** Select all that apply.

1. Insecticides
2. Herbicides
3. Fungicides
4. Heavy metals
5. Rodenticides
6. Microorganism
7. Human / animal waste
8. Others (specify)

**Q3. Do you believe that humans can get diseases from:** Check all that you believe to be true.

1. Animals
2. Plants
3. Environment
4. None

**Q4. Which of the following do you believe to be true?**

1. Animals can transmit diseases to humans.
2. Humans can transmit diseases to animals.
3. The water quality can affect human health.
4. If you can see through water (it is clear) that means it is safe to drink.
5. How we fertilize vegetables and fruits can affect human health.
6. The quality of water can cause disease in animals.
7. Antibiotic use in animals can affect human health.
8. Butchering animals in your backyard can affect human health.

**Q5. Which of the following changes to the environment can affect human and animal health?**

1. Cutting down forests
2. Building homes in rural areas that were previously only inhabited by wildlife
3. A rise in air temperature
4. Mining and landscape changes
5. An increase in the number of mosquitos
6. A decrease in freshwater availability
7. An increase in standing water after flooding

**F. FAMILY-LEVEL HEALTH PRACTICES**

**Q1. Which of the following do you practice in your household?** Select all that apply.

1. Cook your food to the proper temperature
2. Wash your hand and cooking surfaces before and after preparing your food.
3. Separate raw meat from fruit and vegetables

**Q2. Which of the following has your family practiced in the past year?** Check all that you believe to be true.

1. Eaten uncooked meat
2. Eaten uncooked eggs
3. Consumed unpasteurized milk or cheese
4. Eaten animals that you find dead
5. Culled / killed sick livestock / poultry for consumption
6. Washed hands with soap after handling animals
7. Kept animals away from food preparation areas
8. Fertilized crops with raw manure
9. Hunted and/or slaughtered wild animals
